# Closed-loop vasopressor systems for hemodynamic control in perioperative and critical care settings: a systematic review and meta-analysis

**DOI:** 10.1007/s10877-026-01463-7

**Published:** 2026-07-22

**Authors:** Jorge Iván Alvarado-Sánchez, Diana María Abril-Calderón, Isabella Sánchez-Quimbayo, Lucas Oñoro-Patiño, Camilo Vega-Useche, Juan Sebastián Reyes-Gonzales, Fredy Leonardo Carreño-Hernandez

**Affiliations:** 1 Department of Critical Medicine and Intensive Care, Fundación Santa Fe de, Bogotá, Colombia; 2 Department of Physiology Sciences, Faculty of Medicine, Universidad Nacional de Colombia, Bogotá, Colombia; 3 School of Medicine, Universidad del Rosario, Bogotá, Colombia; 4 School of Medicine, Universidad de los Andes, Bogotá, Colombia

**Keywords:** Closed-loop control, Haemodynamic management, Hypotension, Meta-analysis, Norepinephrine, Perioperative medicine, Vasopressors

## Abstract

Maintaining mean arterial pressure (MAP) within a predefined target is central to haemodynamic management in surgical and critically ill adults receiving vasopressors. Closed-loop vasopressor (CLV) systems automate titration to optimise blood pressure control, but their clinical effectiveness remains uncertain. We performed a systematic review and meta-analysis comparing CLV with manual titration. This PRISMA 2020–compliant review was prospectively registered in PROSPERO (CRD420250655697). MEDLINE, Embase, Scopus, Web of Science, CENTRAL, and the Cochrane Library were searched (January 2000–June 2025). Randomised controlled trials enrolling adults receiving vasopressors in perioperative or intensive care settings were included. Primary outcomes were time within the MAP target range and time spent in hypotension or hypertension. Risk of bias was assessed using RoB 2.0 and certainty of evidence using GRADE. Random- or fixed-effects models were selected according to heterogeneity. Six randomized controlled trials (215 patients) were included in the systematic review, whereas five perioperative trials contributed to the meta-analysis of haemodynamic control outcomes, and one ICU-based study was summarized narratively because it did not report comparable MAP control endpoints. CLV increased time within the MAP target range (mean difference [MD] 33.94%, 95% CI 20.41–47.46; I^2^ = 77%) and reduced time in hypotension (MD − 18.24%, 95% CI − 28.95 to − 7.53; I^2^ = 73%). There was no significant difference in time in hypertension, cumulative norepinephrine dose, or major/minor adverse events. ICU length of stay was not pooled because of clinical and methodological heterogeneity. Certainty of evidence ranged from low to high (moderate for haemodynamic control outcomes). CLV systems improved haemodynamic control, primarily in perioperative settings, but heterogeneity and small samples limit confidence in effect size and generalisability. Evidence in critically ill populations remains limited, and larger trials are needed to determine whether improvements in these physiological surrogate endpoints translate into meaningful patient-centred outcomes.

**Supplementary Information:**

The online version contains supplementary material available at https://doi.org/10.1007/s10877-026-01463-7.

## Introduction

Maintaining arterial pressure within an adequate range is a central objective of haemodynamic management in perioperative and critical care settings [1–4]. Even brief episodes of hypotension are associated with acute kidney injury, myocardial injury, and increased mortality, while excessive hypertension may also be harmful by increasing myocardial oxygen demand and bleeding risk [4–7].

Vasopressors, most commonly norepinephrine, are the primary strategy for achieving target mean arterial pressure (MAP) [8–11]. However, their administration relies on manual titration, which is intermittent, reactive, and operator-dependent. As a result, blood pressure control is often suboptimal, with patients spending substantial time outside predefined targets, highlighting a mismatch between continuous physiology and human-driven interventions.

Closed-loop vasopressor systems aim to address this limitation by integrating continuous blood pressure monitoring with automated control algorithms that adjust infusion rates in real time to maintain MAP within a predefined target range. Early clinical studies suggest that these systems can increase time within target and reduce hypotensive exposure [12, 13]. However, available evidence remains limited to small, short-duration trials conducted in controlled environments, and it remains unclear whether improved haemodynamic precision translates into clinically meaningful benefits [12, 13].

We conducted a systematic review and meta-analysis to evaluate whether closed-loop vasopressor systems improve haemodynamic control—specifically time within target MAP and exposure to hypotension and hypertension—as well as vasopressor requirements and clinical outcomes.

## Methods

### Protocol

This systematic review and meta-analysis was conducted in accordance with the Preferred Reporting Items for Systematic Reviews and Meta-Analyses (PRISMA) 2020 statement and was prospectively registered in the International Prospective Register of Systematic Reviews (PROSPERO; https://www.crd.york.ac.uk/PROSPERO/view/CRD420250655697**) **[14].

### Search strategy and data extraction

MEDLINE (via PubMed), Embase, Scopus, Web of Science, the Cochrane Central Register of Controlled Trials (CENTRAL), and Cochrane Library were systematically searched for peer-reviewed articles from January 1, 2000, to June 30, 2025, without language restrictions. The search strategy combined controlled vocabulary (e.g., MeSH and Emtree terms) and free-text keywords related to closed-loop systems, automated vasopressor titration, norepinephrine administration, and blood pressure control in surgical and critically ill patients. The complete electronic search strategies for all databases, including all search terms and Boolean operators, are provided in Supplementary Appendix 1 and were prospectively predefined in the registered study protocol (PROSPERO; https://www.crd.york.ac.uk/PROSPERO/view/CRD420250655697).

Three authors independently screened the retrieved records and assessed full texts for eligibility according to the predefined inclusion and exclusion criteria (D.M.A.C., I.S.Q. and L.O.P); disagreements were resolved through discussion and, when necessary, by consensus with a fourth author (J.I.A.S). In addition, reference lists of all eligible studies and relevant reviews were manually screened to identify potentially eligible trials not captured in the database search. The detailed search strategy, including keywords and index terms, was predefined in the study protocol and prospectively registered in PROSPERO (CRD420250655697; https://www.crd.york.ac.uk/PROSPERO/view/CRD420250655697).

### Study selection and inclusion criteria

Studies were selected according to the predefined eligibility criteria based on the clinical question framework as follows:

- Population: We included randomized controlled trials enrolling adult patients (≥ 18 years) in perioperative or critical care (ICU) settings who required vasopressor therapy for blood pressure support.
- Intervention: Studies evaluating closed-loop vasopressor administration systems, defined as automated titration strategies capable of adjusting vasopressor delivery in real time based on continuous or near-continuous blood pressure monitoring with the objective of maintaining a predefined mean arterial pressure (MAP) target.
- Comparator: Studies in which the control group received manual vasopressor titration, performed by clinicians according to standard practice (e.g., adjustment of infusion rates or bolus administration based on clinical judgment and monitoring data).
- Outcomes: Eligible studies reported at least one prespecified outcome related to haemodynamic control, including % time within the target MAP range, % time in hypotension, and/or % time in hypertension. Secondary outcomes of interest included cumulative vasopressor dose, ICU length of stay, and adverse events, when available.
- Studies: Only trials with a direct comparison between closed-loop systems and manual titration were included. Studies not reporting MAP-based endpoints, non-randomized designs, pediatric populations, simulator studies, and animal studies were excluded.

### Exclusion criteria

Studies were excluded if they enrolled patients younger than 18 years, were non-randomized designs (e.g., case reports, case series, observational studies), were published only as conference abstracts, or involved animal experiments. Obstetric studies evaluating spinal anaesthesia–related hypotension during caesarean delivery were also excluded because they represent a physiologically distinct scenario predominantly managed with phenylephrine or ephedrine bolus administration rather than continuous vasopressor infusion control, thereby limiting comparability with perioperative and critically ill adult populations.

### Study selection and data collection

Three authors (D.M.A.C., I.S.Q., and L.O.P.) independently extracted data from all included studies using separate standardized extraction spreadsheets. The independently completed extraction forms were subsequently compared to ensure consistency and accuracy. Discrepancies were initially resolved through discussion among the reviewers. When disagreements persisted, a fourth author (J.I.A.S.) reviewed the extraction forms and adjudicated to reach consensus.

### Data items

The data extracted from each eligible randomized controlled trial included study characteristics (first author, year of publication, study design, and sample size), population characteristics (age, clinical diagnosis, severity of illness when reported, and surgical procedure or ICU setting), intervention details (type of closed-loop vasopressor system and vasopressor agent used), and comparator details (manual titration approach). Extracted outcomes comprised the prespecified endpoints related to haemodynamic control, including time within the target mean arterial pressure (MAP) range, time in hypotension, and time in hypertension, as well as secondary outcomes such as cumulative vasopressor dose, ICU length of stay, and adverse events. When outcomes were reported at multiple time points or across different observation windows, data were extracted according to the definitions provided in each trial and categorized consistently with the prespecified outcomes of this review.

### Risk of bias in individual studies

Risk of bias was assessed using the Cochrane Risk of Bias tool (RoB 2.0) for randomized controlled trials [15]. Three independent reviewers (D.M.A.C., I.S.Q., and L.O.P.) evaluated the following domains: (1) randomization process, (2) deviations from intended interventions, (3) missing outcome data, (4) measurement of the outcome, and (5) selection of the reported result. Discrepancies were resolved through discussion and, when necessary, by consultation with a fourth reviewer (J.I.A.S.) to reach consensus.

### Statistical analysis

#### Analysis of individual studies and effect measures

For each included randomized controlled trial, effect estimates were calculated according to outcome type. Dichotomous outcomes (e.g., incidence of hypotension, adverse events) were summarized using risk ratios (RR) with 95% confidence intervals (CIs). Continuous outcomes (e.g., time within the target MAP range, vasopressor consumption, ICU length of stay) were summarized using mean differences (MD) with 95% CIs when outcomes were reported on comparable scales; otherwise, standardized mean differences (SMD) were used. When continuous data were reported as medians with interquartile ranges (IQRs), they were converted to means and standard deviations (SDs) using established statistical methods to enable quantitative synthesis [16]. Random-effects meta-analyses were performed using the restricted maximum likelihood (REML) estimator, and Hartung–Knapp adjustments were applied when random-effects models were used. Prediction intervals were calculated to estimate the expected range of true effects in future comparable studies. No imputation of missing SDs was performed beyond conversions derived from reported summary statistics.

#### Analysis of summary measures and meta-analysis

When at least two studies reported the same outcome in a comparable manner, pooled estimates were obtained through meta-analysis. Statistical heterogeneity was evaluated using Cochran’s Q test, and its magnitude was quantified using the I^2^ statistic. We used a fixed-effects model when statistical heterogeneity was low (I^2^ < 50%). When heterogeneity was moderate to substantial (I^2^ ≥ 50%), we applied a random-effects model. Statistical significance was defined as a two-sided p-value < 0.05.

#### Analysis of risk of bias across studies

Publication bias was not formally assessed because the number of included studies was insufficient to provide reliable funnel plot or Egger’s test estimates. However, given the expected limited number of included trials for some outcomes, statistical tests for publication bias were interpreted cautiously.

#### Additional analyses

Sensitivity analyses were planned by excluding studies judged to have a high risk of bias. Prespecified subgroup analyses were planned based on clinically relevant factors, including vasopressor type, surgical procedure, severity of illness, and clinical setting (ICU vs perioperative environment).

All statistical analyses were performed using R software, primarily using the meta and metafor packages for quantitative synthesis and meta-analysis.

## Results

**Fig. 1:**
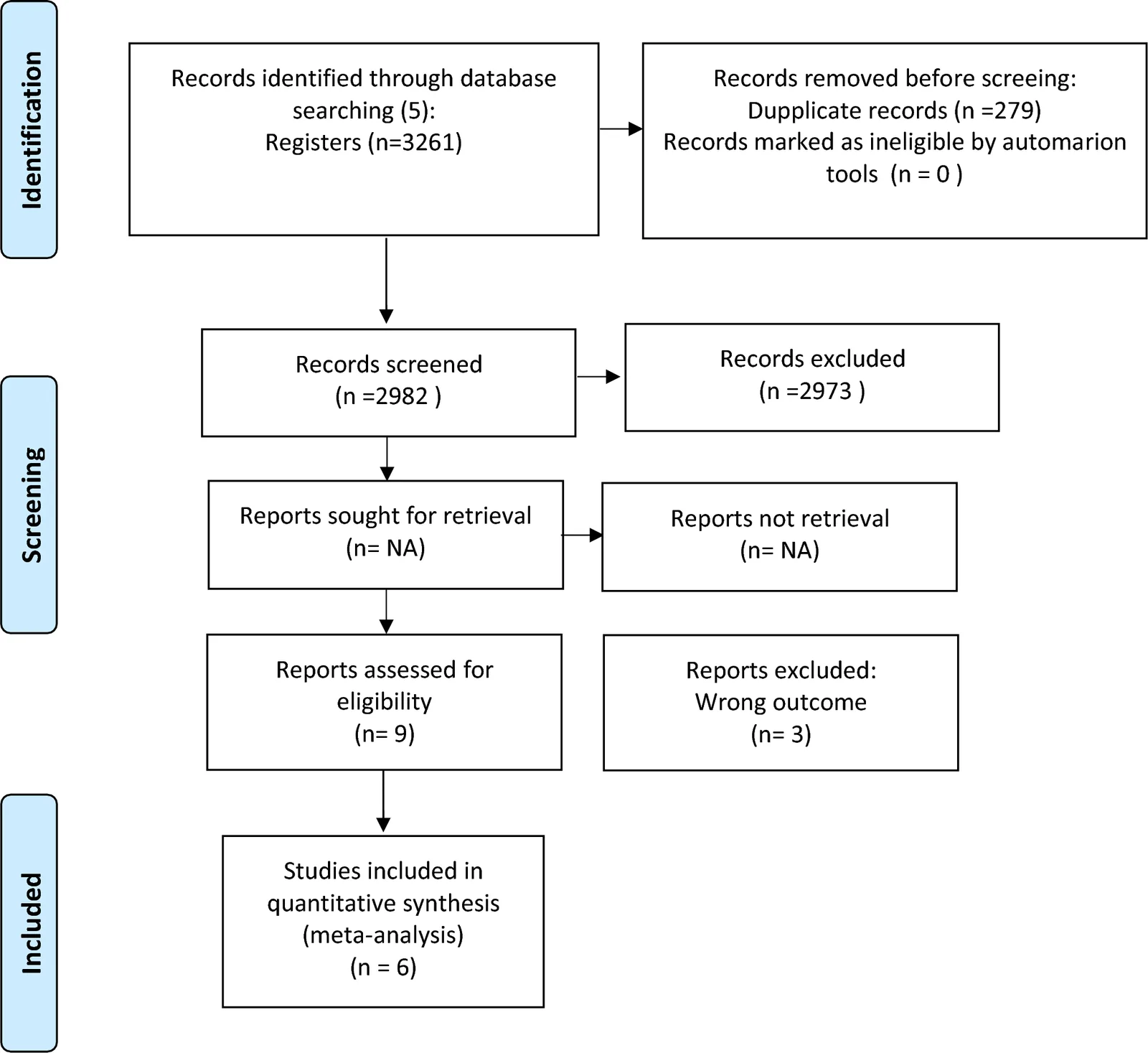
PRISMA 2020 flow diagram of study identification, screening, eligibility assessment, and inclusion in the systematic review and meta-analysis

A total of 3,261 records were identified through database searching across six databases. After removal of 279 duplicates, 2,982 records were screened by title and abstract, of which 2,973 were excluded. Nine full-text articles were assessed for eligibility [17–25]. Of these, three were excluded because they did not report the prespecified outcomes for meta-analysis. Consequently, six randomized controlled trials were included in the quantitative synthesis (Fig. 1) [18, 19, 21, 23–25].

### Characteristics of included studies

Six randomized controlled trials published between 2008 and 2024 were included, comprising 215 participants. Sample sizes ranged from 18 to 53 patients, and all studies compared a CLV system with manual titration strategies [18, 19, 21, 23–25].

The trials were conducted across heterogeneous clinical settings, predominantly in perioperative environments, including intermediate- to high-risk noncardiac surgery and post–cardiac or abdominal surgery, with only one study performed in an intensive care unit setting (septic shock during norepinephrine).

Closed-loop systems incorporated different control architectures—such as fuzzy logic [18], proportional–integral–derivative–based algorithms [19, 21, 23, 25], predictive rule-based controllers [19, 21], and computer-assisted haemodynamic management platforms [23–25]—but all were designed to automatically adjust vasopressor infusion based on continuous arterial pressure measurements.

Reported outcomes primarily focused on blood pressure control metrics, including time within the target range and exposure to hypotension, as well as cumulative norepinephrine dose and adverse events [19, 21, 23–25]. Some studies additionally reported healthcare utilization outcomes, such as intensive care unit length of stay, while one trial evaluated survival at 28 days [18].

Observation periods varied according to the clinical context, ranging from intraoperative monitoring to short-term postoperative or intensive care assessments lasting 2 to 4 h, and from vasopressor weaning initiation until drug discontinuation. Key characteristics of the included studies are summarized in Table 1.

**Table 1:** Characteristics of included studies evaluating closed-loop vasopressor systems compared with manual titration strategies across perioperative and intensive care settings

Study	Year	Sample Size (CLV / Control)	Closed-Loop Strategy	Comparator	Population	Key Outcomes	Observation Period
Coeckelenbergh et al	2024	27 / 26	Hybrid predictive rule-based algorithm (PID)	Manual titration by ICU nursing staff	Adults following high-risk abdominal surgery (e.g., liver transplantation)	% time within target (80–90 mmHg), time in hypotension (< 80 and < 65 mmHg)	2 h (immediate postoperative ICU period)
Joosten et al. (COMAT)	2024	6 / 12	Automated norepinephrine titration system	Manual titration by ICU nursing staff	Patients with severe brain injury (Glasgow Coma Scale < 9)	% time within target (± 5 mmHg of goal), time above/below target	4 h (ICU)
Desebbe et al	2022	20 / 17	Closed-loop norepinephrine controller	Manual titration by ICU nursing staff	Post–cardiac surgery patients	% time in hypotension (MAP < 65 mmHg), % time within target (65–75 mmHg)	2 h (ICU)
Joosten et al. (2021a)	2021	19 / 19	Assisted haemodynamic management (closed-loop vasopressor plus automated fluid management)	Goal-directed manual management	Intermediate- to high-risk abdominal or orthopedic surgery	% time in hypotension (MAP < 90% of baseline), minor complications	Intraoperative (induction to end of surgery)
Joosten et al. (2021b)	2021	15 / 15	Closed-loop norepinephrine controller	Standard manual norepinephrine titration	Elective intermediate- to high-risk abdominal surgery	% time in hypotension (MAP < 90% of baseline), time within target	Intraoperative
Merouani et al	2008	19 / 20	Fuzzy logic–based closed-loop control	Clinician-directed management (intensivist discretion)	Patients with septic shock during norepinephrine weaning	Time to norepinephrine discontinuation (shock duration), total norepinephrine dose	Until vasopressor discontinuation or death

Closed-loop systems used automated algorithms—including fuzzy logic, proportional control, predictive rules, and computer-assisted decision support—to adjust vasopressor infusion rates based on continuous arterial pressure monitoring. Comparators consisted of standard manual titration performed by clinicians or protocolized goal-directed therapy. Outcomes primarily assessed haemodynamic control (e.g., time within target mean arterial pressure or systolic blood pressure ranges, incidence of hypotension or hypertension), vasopressor exposure, clinical endpoints (length of stay, survival, complications), and maternal–neonatal variables when applicable. Observation periods varied according to the clinical context, ranging from intraoperative monitoring to short-term postoperative or intensive care unit assessments. CLV = closed-loop vasopressor control; ICU = intensive care unit; MAP = mean arterial pressure; PID = proportional–integral–derivative; GCS = Glasgow Coma Scale; GDT = goal-directed therapy; AFM = automated fluid management; OR = operating room. Targets and outcome definitions are reported as described in the original studies

### Risk of bias

**Fig. 2:**
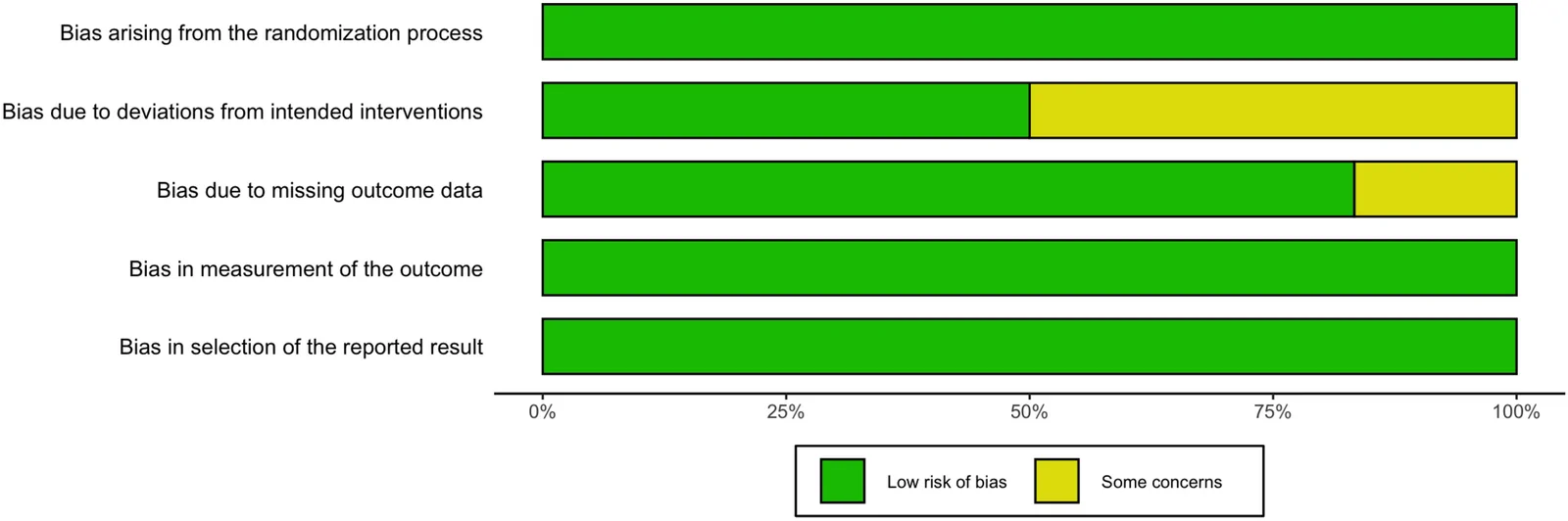
Risk of bias summary of included randomized controlled trials assessed with the Cochrane Risk of Bias tool (RoB 2.0)

Of the six included trials, three were judged as having an overall low risk of bias [18, 19, 21], whereas the remaining three were rated as having some concerns [23–25]; no study was classified as high risk (Fig. 2 and Table 2).

**Table 2:** Risk of bias assessment of included randomized trials using the Cochrane Risk of Bias 2 (RoB 2) tool

Study	D1: Randomization Process	D2: Deviations from Intended Interventions (Blinding)	D3: Missing Outcome Data	D4: Outcome Measurement	D5: Selection of Reported Results	Overall Risk
Merouani (2008)	Low risk	Low risk (blinded outcome assessors)	Low risk (intention-to-treat analysis)	Low risk (objective measurement)	Low risk (registered protocol)	Low risk
Joosten (2021a)	Low risk	Some concerns (care providers not blinded)	Low risk (no missing data)	Low risk (automated EV1000 monitoring)	Low risk (registered protocol)	Some concerns
Joosten (2021b)	Low risk	Some concerns (care providers not blinded)	Low risk	Low risk (automated EV1000 monitoring)	Low risk (registered protocol)	Some concerns
Desebbe (2022)	Low risk	Low risk (blinding maintained during surgery)	Low risk	Low risk (automated EV1000 monitoring)	Low risk (registered protocol)	Low risk
Coeckelenbergh (2024)	Low risk	Low risk (clinicians and outcome assessors blinded)	Low risk	Low risk (automated EV1000 monitoring)	Low risk (registered protocol)	Low risk
Joosten (2024, COMAT)	Low risk	Some concerns (personnel not blinded)	Some concerns (high attrition due to refusals)	Low risk	Low risk (registered protocol)	Some concerns

The RoB 2 framework evaluates five domains: bias arising from the randomization process (D1), bias due to deviations from intended interventions, including blinding (D2), bias due to missing outcome data (D3), bias in outcome measurement (D4), and bias in the selection of reported results (D5). Each domain was judged as *low risk*, *some concerns*, or *high risk*, leading to an overall risk-of-bias judgment for each study. RoB 2, revised Cochrane risk-of-bias tool for randomized trials; EV1000, Edwards Lifesciences haemodynamic monitoring platform

Concerns were primarily related to deviations from intended interventions due to lack of blinding of care providers, and in one trial [24], to missing outcome data associated with higher attrition. The remaining domains—including the randomization process, outcome measurement, and selection of reported results—were consistently judged as low risk across studies.

Using the GRADE framework, the certainty of evidence for the primary outcomes ranged from low to high. Certainty was moderate for time within MAP target, time spent in hypotension or hypertension, and major adverse events, low for cumulative norepinephrine dose, and high for minor adverse events (Supplementary Material, Table 1).

### Haemodynamic control

Because fewer than six studies were included in each analysis, publication bias was not formally assessed using funnel plots.

Five randomized controlled trials involving 176 patients evaluated different metrics of haemodynamic control [19, 21, 23–25].

**Fig. 3:**
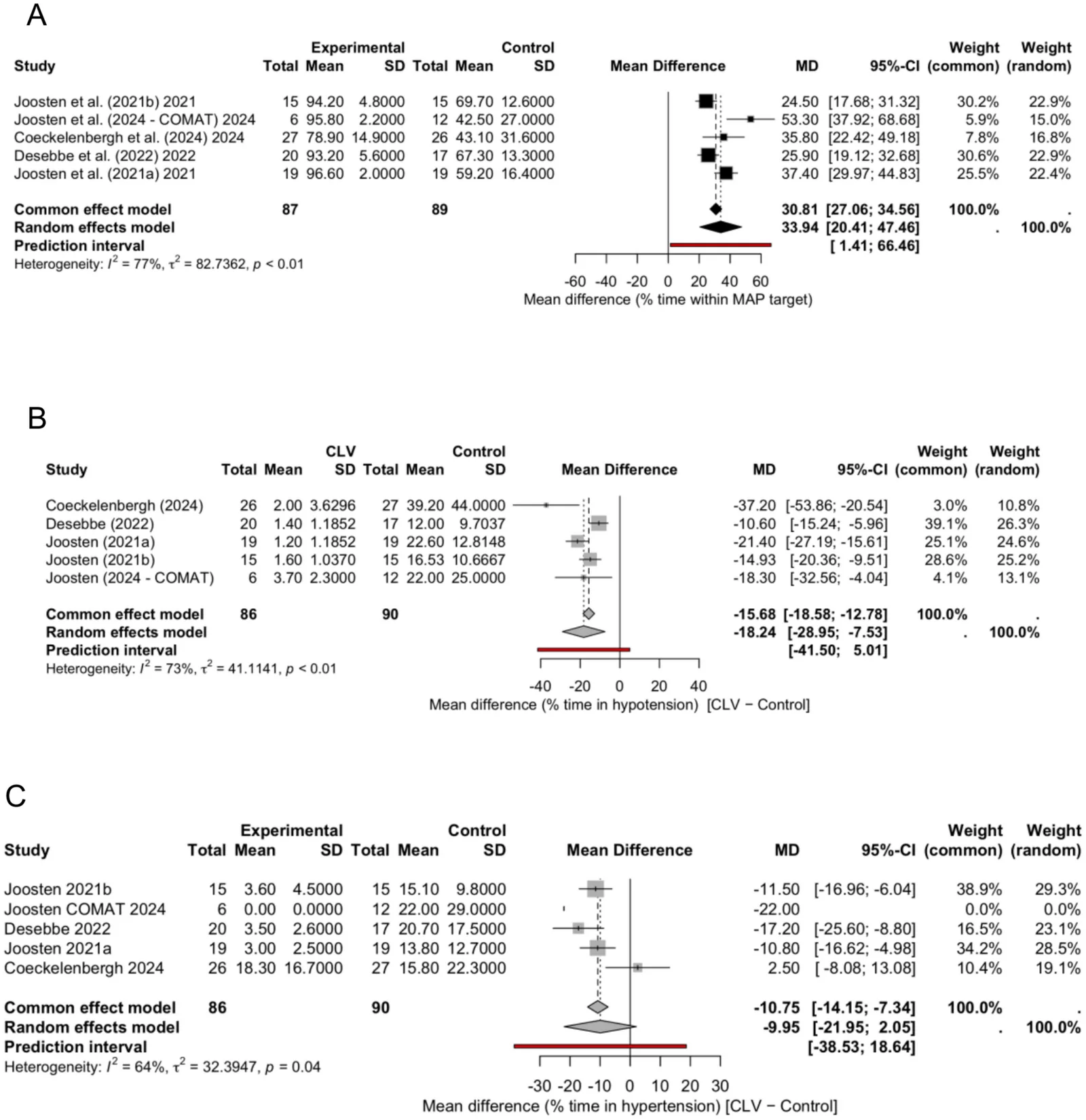
Forest plots comparing closed-loop vasopressor systems versus manual titration for haemodynamic control outcomes: (**A**) percentage of time within the predefined mean arterial pressure (MAP) target range, (**B**) percentage of time spent in hypotension, and (**C**) percentage of time spent in hypertension; effects are presented as mean differences with 95% confidence intervals using random-effects models

Closed-loop vasopressor administration significantly increased the percentage of time within the predefined MAP target compared with manual titration (random-effects mean difference [MD] 33.94%, 95% CI 20.41 to 47.46; I^2^ = 77%) (Fig. 3a).

Closed-loop control was also associated with a reduction in the proportion of time spent in hypotension (MD − 18.24%, 95% CI − 28.95 to − 7.53; I^2^ = 73%) (Fig. 3b).

For time in hypertension, the pooled estimate favored closed-loop titration but did not reach statistical significance (MD − 9.95%, 95% CI − 21.95 to 2.05), with moderate heterogeneity (I^2^ = 64%) (Fig. 3c).

Prediction intervals were wide across analyses, indicating variability in the estimated effects.

All pooled haemodynamic control outcomes were derived exclusively from perioperative studies, as the only ICU-based trial did not report comparable endpoints.

### Secondary outcomes

#### Cumulative norepinephrine dose

**Fig. 4:**
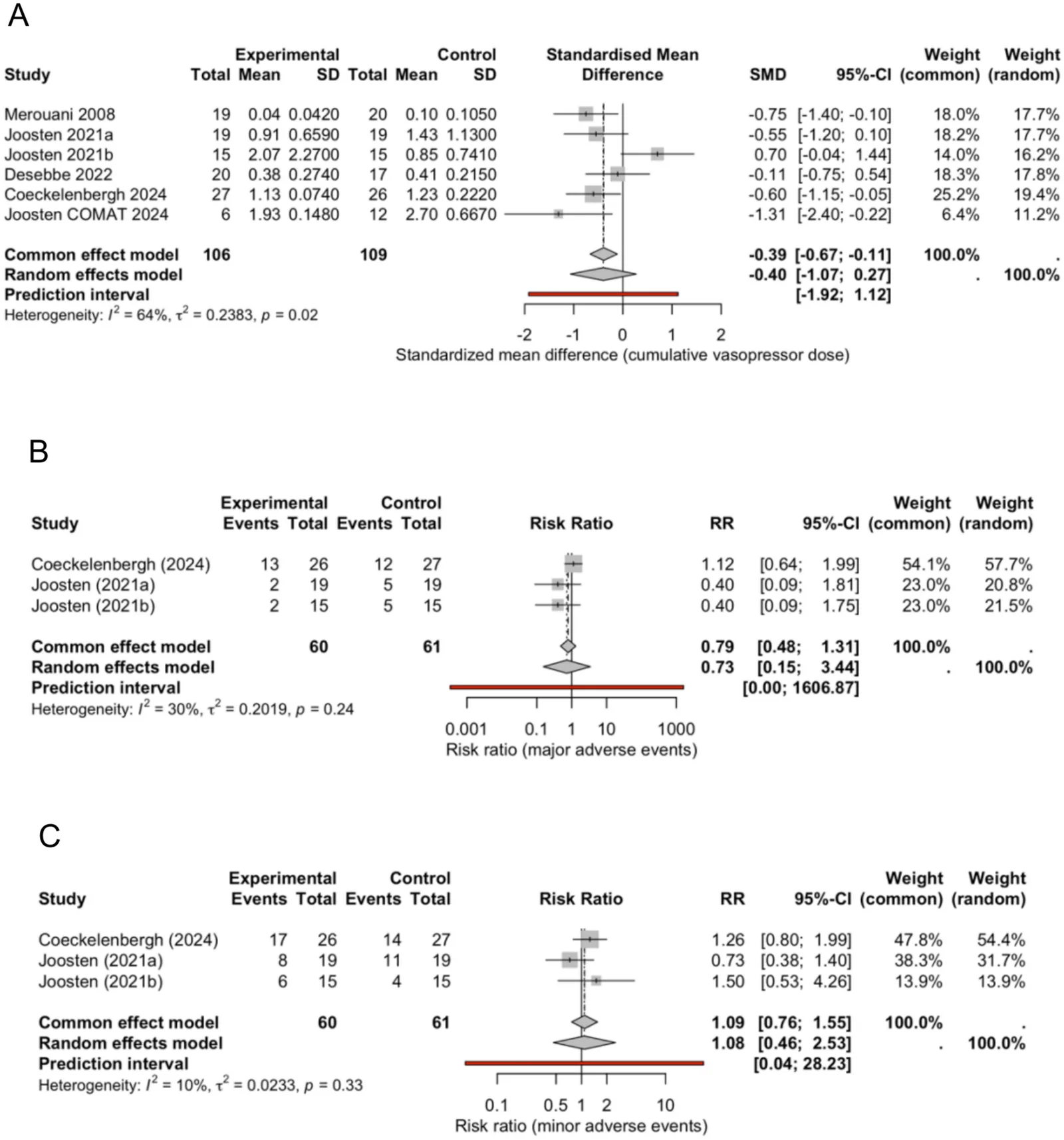
Forest plots comparing closed-loop vasopressor systems versus manual titration for secondary outcomes: (**A**) cumulative norepinephrine dose expressed as standardized mean difference, (**B**) major adverse events, and (**C**) minor adverse events; effects are presented with 95% confidence intervals (risk ratios for adverse events)

Six randomized controlled trials reported cumulative norepinephrine dose[18, 19, 21, 23–25]. The pooled analysis, performed using standardized mean differences, showed no statistically significant difference between closed-loop vasopressor administration and manual titration (SMD − 0.40, 95% CI − 1.07 to 0.27; I^2^ = 64%). The prediction interval ranged from − 1.92 to 1.12 (Fig. 4a).

#### Adverse events

Major adverse events were reported in three trials [19, 23, 25]. The pooled analysis showed no statistically significant difference between groups (risk ratio [RR] 0.73, 95% CI 0.15 to 3.44; I^2^ = 30%), with a prediction interval of 0.00 to 1606.87 (Fig. 4b).

Minor adverse events were also reported in three studies [19, 23, 25], with no statistically significant difference observed between closed-loop and manual strategies (RR 1.08, 95% CI 0.46 to 2.53; I^2^ = 10%). The prediction interval ranged from 0.04 to 28.23 (Fig. 4c).

#### Intensive care unit length of stay

Several studies reported ICU length of stay as a secondary or exploratory outcome[18, 19, 21, 23, 25]; however, a quantitative synthesis was not performed due to substantial clinical and methodological heterogeneity, including differences in reporting units (hours vs. days), outcome definitions, and the use of alternative metrics such as ICU-free days.

Across studies, no statistically significant differences were reported between groups. Coeckelenbergh and colleagues observed ICU stays of 68 [38–121] hours in the closed-loop group and 86 [44–117] hours in the control group (P = 0.522) [19]. Joosten and colleagues reported combined PACU/ICU stays of 9.0 [5.0–24.0] versus 9.0 [4.0–24.0] hours (P = 0.988) [25], while Joosten and colleagues reported identical durations of 19 [18–20] hours (P = 0.576)[23]. Desebbe and colleagues similarly found no between-group differences in ICU or hospital stay [21].

Merouani and colleagues, conducted in patients with septic shock, reported ICU-free days at day 28 rather than absolute length of stay (3 [0–17] vs. 7 [0–18] days; P = 0.74) [18]. Joosten and colleagues did not report ICU length of stay [23].

### Additional results

The planned sensitivity analyses were not performed due to the limited number of included studies and the small overall sample size, which precluded meaningful subgroup comparisons and robust re-estimation of pooled effects.

## Discussion

Closed-loop vasopressor systems were associated with improved physiological haemodynamic control compared with manual titration, primarily by maintaining arterial pressure within predefined targets and reducing hypotensive exposure without increasing vasopressor requirements or adverse events. Importantly, these findings are entirely driven by perioperative studies, as the pooled haemodynamic control outcomes were derived exclusively from trials conducted in surgical settings, and the only ICU-based study—focused on vasopressor weaning—did not report comparable haemodynamic control endpoints and therefore did not contribute to the pooled estimates. However, these improvements in time within the target MAP range and reduction in hypotensive exposure represent physiological surrogate endpoints rather than direct clinical outcomes and therefore should not be interpreted as evidence of improved patient-centred benefit. Consistently, no significant differences were observed in clinically meaningful outcomes such as cumulative vasopressor dose, adverse events, or ICU length of stay, although this may reflect either limited statistical power or a true absence of clinically relevant effect. Collectively, these findings suggest that automation primarily enhances the precision and consistency of vasopressor delivery rather than producing demonstrated improvements in clinical outcomes.

Our findings align with prior meta-analyses evaluating automated drug delivery. The umbrella review by Spataru and colleagues included four randomized trials assessing norepinephrine and showed that closed-loop systems reduced the duration of blood pressure outside prespecified targets, supporting the physiological advantage of automated feedback [12]. Similarly, the obstetric meta-analysis by Khan and colleagues demonstrated lower hypotension incidence and attenuation of extreme systolic blood pressure values with closed-loop control. Across these syntheses—and consistent with our results—automation did not significantly reduce cumulative vasopressor dose, reinforcing the interpretation that its benefit lies in delivery efficiency rather than drug sparing [13].

More recently, Chiou and colleagues conducted a systematic review and meta-analysis including eight randomized controlled trials and 640 patients comparing closed-loop vasopressor systems with manual titration. Consistent with our findings, they demonstrated improved haemodynamic precision, including greater time spent within target blood pressure ranges and reduced hypotensive exposure, without a significant reduction in total vasopressor consumption [26].

Important differences, however, underscore the added value of the present meta-analysis. While the prior norepinephrine synthesis incorporated four randomized trials [12], our study includes six randomized controlled trials, expanding the evidence base and integrating more contemporary data. Moreover, the obstetric analysis focused exclusively on caesarean delivery under spinal anaesthesia, a physiologically distinct scenario characterized by sympathectomy-driven hypotension, which limits extrapolation to critically ill or general surgical populations [13]. For this reason, obstetric trials were not included in our analysis, as their distinct physiological and haemodynamic context would limit comparability with non-obstetric populations. Similarly, although the recent meta-analysis by Chiou et al. included a larger overall sample size, a substantial proportion of participants were derived from obstetric studies, which may limit the applicability of its findings to non-obstetric populations. By deliberately restricting our analysis to non-obstetric adults, we sought to provide a more clinically homogeneous assessment of automated vasopressor therapy in perioperative and critical care settings [26]. Finally, broader reviews assessed multiple pharmacologic classes—including sedatives, insulin, and neuromuscular blockers—offering conceptual breadth but diluting drug-specific conclusions; our work instead provides a more in-depth [12], clinically oriented evaluation centered specifically on vasopressor-mediated blood pressure control.

Several limitations should be acknowledged. Statistical heterogeneity was substantial across primary analyses, likely reflecting differences in controller algorithms, monitoring strategies, MAP targets, clinical settings, and observation windows. This substantial statistical heterogeneity (reflected by high I^2^ values) may reduce confidence in the pooled effect estimates and suggests that the observed effects may be context-dependent. These variations may limit the comparability of interventions, as different closed-loop systems represent heterogeneous implementations rather than a single uniform strategy. The certainty of evidence was not uniform and ranged from low to high across outcomes. In particular, the low certainty for cumulative norepinephrine dose and the moderate certainty for haemodynamic control outcomes indicate that future adequately powered trials may substantially influence the magnitude and even the direction of some effect estimates. In addition, the relatively small overall sample size (215 patients across six trials) limits statistical power, particularly for safety and patient-centred outcomes. Additionally, several studies were unblinded, and although blinding is inherently challenging in automation-based interventions, the absence of blinding may increase the risk of performance bias. Although some sensitivity analyses were prespecified a priori, they could not be performed because of the limited number of included studies. In some trials, closed-loop vasopressor control was combined with additional automated interventions (e.g., fluid management), which may introduce confounding and limit the ability to isolate the independent effect of vasopressor automation. Moreover, most studies evaluated short intervention periods (typically 2–4 h) within highly controlled perioperative environments, which may overestimate efficacy and limit extrapolation to longer-term or more complex real-world ICU settings. Overall, these factors may limit the external validity and generalisability of the findings.

In conclusion, closed-loop vasopressor systems improve short-term physiological haemodynamic control and reduce hypotensive exposure compared with manual titration, primarily in perioperative settings. However, current evidence is based on small trials, heterogeneous control systems, and surrogate physiological endpoints, which limits confidence in the generalisability and clinical implications of these findings. Larger pragmatic trials are needed to determine whether improved haemodynamic precision translates into meaningful reductions in postoperative or ICU-related complications.

## Supplementary Information

Below is the link to the electronic supplementary material.

Supplementary file 1.

## Data Availability

All data analysed in this study are derived from published articles included in the systematic review. Extracted data and analytic code are available from the corresponding author upon reasonable request.
