# Supplementary material for: Closed-loop vasopressor systems for hemodynamic control in perioperative and critical care settings: a systematic review and meta-analysis
Source: J Clin Monit Comput. 2026 Jul 22;40(5):1065–76. doi: 10.1007/s10877-026-01463-7 (PMC13562163; doi:10.1007/s10877-026-01463-7)
Supplement: Supplementary file 1 [file 10877_2026_1463_MOESM1_ESM.docx]

**Supplementary Appendix 1. Detailed Electronic Search Strategy**

MEDLINE (via PubMed)

Search performed on June 30, 2025.

((((("Vasopressor Agents"[Mesh]) OR "Vasoconstrictor Agents"[Mesh]) AND (("Drug Infusion Systems"[Mesh]) OR ("Closed-Loop Systems"[Mesh]))) AND (("Intensive Care Units"[Mesh]) OR ("Critical Care"[Mesh]) OR ("Surgical Procedures, Operative"[Mesh]) OR ("Perioperative Care"[Mesh]))) AND (("Hypotension"[Mesh]) OR ("Shock, Septic"[Mesh]) OR ("Hemodynamic Monitoring"[Mesh]))) AND (("Length of Stay"[Mesh]) OR ("Postoperative Complications"[Mesh]) OR ("Intraoperative Care"[Mesh]))

Equivalent search strategies adapted to the syntax and controlled vocabulary of Embase, Scopus, Web of Science, CENTRAL, and Cochrane Library were also applied.

Author(s): Jorge Alvarado

Question: Closed-loop vasopressor administration compared to manual titration for haemodynamic control

Setting: in perioperative or critical care

Bibliography:

| Certainty assessment | | | | | | | № of patients | | Effect | | Certainty | Importance |
| --- | --- | --- | --- | --- | --- | --- | --- | --- | --- | --- | --- | --- |
| № of studies | Study design | Risk of bias | Inconsistency | Indirectness | Imprecision | Other considerations | Closed-loop vasopressor administration | manual titration | Relative (95% CI) | Absolute (95% CI) |  |  |
| % of time that MAP was in the target | | | | | | | | | | | | |
| 5 | randomised trials | not serious | not serious | not serious | serious^a^ | none | 87 | 90 | - | mean 33.94 % time within MAP target higher (20.41 higher to 47.46 higher) | ⨁⨁⨁◯ Moderate^a^ | IMPORTANT |
| Percentage time on hypotension | | | | | | | | | | | | |
| 5 | randomised trials | not serious | not serious | not serious | serious^a^ | none | 86 | 90 | - | MD 18.24 % of time with hypotension lower (28.95 lower to 7.53 lower) | ⨁⨁⨁◯ Moderate^a^ | IMPORTANT |
| Percentage time on hypertension | | | | | | | | | | | | |
| 5 | randomised trials | not serious | not serious | not serious | serious^a^ | none | 86 | 90 | - | MD 9.95 % of time in hypertension lower (21.95 lower to 2.05 higher) | ⨁⨁⨁◯ Moderate^a^ | IMPORTANT |
| Cumulative norepinephrine dose | | | | | | | | | | | | |
| 6 | randomised trials | not serious | serious^b^ | not serious | serious^a^ | none | 106 | 109 | - | MD 0.4 mg lower (1.07 lower to 0.27 higher) | ⨁⨁◯◯ Low^a,b^ | IMPORTANT |
| Major adverse event | | | | | | | | | | | | |
| 3 | randomised trials | not serious | not serious | not serious | not serious | none | 17/60 (28.3%) | 22/61 (36.1%) | RR 0.73 (0.15 to 3.44) | 97 fewer per 1000 (from 307 fewer to 880 more) | ⨁⨁⨁◯ Moderate | IMPORTANT |
| Minor adverse event | | | | | | | | | | | | |
| 3 | randomised trials | not serious | not serious | not serious | not serious | none | 31/60 (51.7%) | 29/61 (47.5%) | RR 1.08 (0.46 to 2.53) | 38 more per 1000 (from 257 fewer to 727 more) | ⨁⨁⨁⨁ High | IMPORTANT |

CI: confidence interval; MD: mean difference; RR: risk ratio

#### Explanations

a. Heterogeneity could not be assessed

b. There are studies with opposing directions of effect.
